# Reply to the Comment: Comparing ChatGPT and Dental Students' Performance in an Introduction to Dental Anatomy Examination

**DOI:** 10.1055/s-0046-1820115

**Published:** 2026-06-03

**Authors:** Rizwan Ullah, Muhammad Saad Shaikh, Muhammad Sohail Zafar

**Affiliations:** 1 Department of Oral Biology, Sindh Institute of Oral Health Sciences, Jinnah Sindh Medical University, Karachi, Pakistan; 2 Clinical Sciences Department, College of Dentistry, Ajman University, Ajman, United Arab Emirates; 3 Centre of Medical and Bio-allied Health Sciences Research, Ajman University, Ajman, United Arab Emirates; 4 School of Dentistry, University of Jordan, Amman, Jordan

The authors would like to thank Hinpetch Daungsupawong and Viroj Wiwanitkit for providing constructive feedback and valuable insights in their letter to the editor, 1 regarding our original cross-sectional study on the comparison of ChatGPT and Dental Students' performance for a dental anatomy examination. 2 In this letter, the authors appreciated the study 2 and raised some comments that we would like to address. Cross-sectional studies, whether analytical or descriptive, are snapshot studies and are not suitable for long-term comparisons; therefore, for long-term analysis, cohort studies are preferred, which typically include follow-up. Since our study population consists of first-year dentistry students who have just been admitted to the dental school, we also reported the prior educational backgrounds of the admitted students in our paper. Considering this, our test questions focused on basic dental anatomy rather than clinical reasoning or knowledge application.

The primary focus was likely a direct comparison of correct/incorrect answers, for which frequencies are highly relevant. In our study, we utilized the Shapiro–Wilk test and the Kolmogorov test and calculated Cronbach's *α* value to determine the reliability of the items included in our test. Therefore, in our opinion, the selection of the software and the tests is appropriate.

We agree with the comments regarding ChatGPT's accuracy and lower reliability, indicating that AI language models' inability to perform in-depth data analysis or interpretation, particularly in health science contexts. 1 We agree with the comments and have already discussed this in the discussion section of the article by Ullah et al. 2 Furthermore, we also agree with the authors of the letter regarding the use of other AI models for comparison purposes, and we have discussed this in detail in our study, with recommendations in the latter half of the discussion and conclusion.

Regarding the use of AI in dental education and research, we agree with the author's recommendation and have given similar recommendations in the conclusion section of our study. We fully acknowledge the concern about the potential research comparing ChatGPT with other AI models specifically trained for dental education, including BioGPT. 3 We have addressed the limitations and related future directions in the last paragraph of the discussion. Due to constraints in dental educational settings, exact comparisons between our cohort and other studies are not possible.

The authors would also like to emphasize that adherence to emerging ethical and reporting guidelines is essential to ensure transparency, accountability, and academic integrity when integrating Large Language Models into dental research. By following the reporting guidelines, educators and researchers can decrease the risks of bias and data privacy, promoting responsible generative AI adoption. 4 In addition, as we discussed, ChatGPT and other AI systems are continually evolving due to rapid technological advancements, user feedback, and data pooling. 2 Integration of ChatGPT with relevant knowledge inputs is likely to significantly enhance ChatGPT's performance. 5 Therefore, future studies should focus on exploring the students' feedback on ChatGPT's explanations to provide valuable insights into its practical educational applications.
